# Hypnotic suggestibility as a moderator of treatment response in mild to moderate depression: an exploratory secondary analysis

**DOI:** 10.3389/fmed.2026.1847384

**Published:** 2026-07-02

**Authors:** Julia Siewert, Benno Brinkhaus, Tatjana Tissen-Diabaté, Michael Teut, Nicolas Volz

**Affiliations:** 1 Charité – Universitätsmedizin Berlin, Corporate Member of Freie Universität Berlin and Humboldt-Universität zu Berlin, Institute of Social Medicine, Epidemiology and Health Economics, Berlin, Germany; 2 Brandenburg Medical School Theodor Fontane, Neuruppin, Germany

**Keywords:** creative imagination scale, depressive symptoms, group hypnosis, Harvard group scale of hypnotic susceptibility, suggestibility

## Abstract

**Background:**

Hypnotic suggestibility may moderate treatment outcomes, but evidence in depression is limited and inconsistent.

**Objective:**

This analysis examined whether baseline hypnotic suggestibility moderated changes in depressive symptoms over time and whether this effect differed between groups.

**Methods:**

This exploratory secondary analysis used data from the HypnoDeep trial, a randomized, controlled, pilot trial. Patients with mild to moderate depressive symptoms were randomized to group hypnosis, progressive muscle relaxation (PMR), or control. Hypnotic suggestibility was assessed with the Harvard Group Scale of Hypnotic Susceptibility, Form 5 (HGSHS-5:G) and the Creative Imagination Scale (CIS) at baseline. Depressive symptoms were measured at baseline and at 6 weeks using the Beck Depression Inventory–II. Linear mixed-effects models examined whether baseline suggestibility moderated depressive symptom changes and whether this association differed between groups, adjusting for age and sex. Separate models were estimated for HGSHS-5:G and CIS. A *post hoc* power analysis contextualized detectable effect sizes.

**Results:**

Ninety-four participants were included (mean age 39.2 years, SD 12.0; women: *n* = 67 [71.3%], men: *n* = 25 [26.6%], diverse: *n* = 2 [2.1%]). Baseline hypnotic suggestibility assessed with the HGSHS-5:G ranged from 0 to 5 (mean 2.9, SD 1.6), and CIS scores ranged from 2 to 35 (mean 17.6, SD 7.7). Baseline hypnotic suggestibility was not associated with differential change in depressive symptoms over time in the control group for either the HGSHS-5:G or the CIS, and there was no indication that this association differed in the hypnosis or PMR group compared with the control group (HGSHS-5:G: hypnosis *β* = 2.05, 95% CI −2.21 to 6.31, *p* = 0.350; PMR *β* = −0.97, 95% CI −5.14 to 3.20, *p* = 0.651; CIS: hypnosis *β* = 3.06, 95% CI −1.36 to 7.48, *p* = 0.179; PMR *β* = −0.44, 95% CI −4.49 to 3.61, *p* = 0.833). *Post hoc* power analysis indicated that complete-case group sizes were insufficient to reliably detect small-to-moderate associations.

**Conclusion:**

Exploratory analyses did not indicate that hypnotic suggestibility moderated changes in depressive symptoms across groups. Given limited statistical power, findings are preliminary and hypothesis-generating. Larger studies are needed to clarify treatment-response moderators.

## 1 Introduction

Hypnosis can be conceptualized as a complex, multifaceted mind–body intervention encompassing multiple procedures, phenomena, and influencing factors, with theoretical models highlighting the role of social, contextual, and biopsychosocial mechanisms (1).

Suggestibility denotes the broader propensity to respond to suggestions across both hypnotic and non-hypnotic contexts. This perspective is consistent with response expectancy theory, which posits that responses to suggestions are largely shaped by individuals’ expectancies and do not require the assumption of a distinct altered state of consciousness (2, 3, 33). Theoretical accounts emphasize that responsiveness to suggestion reflects broader cognitive and social processes rather than mechanisms specific to hypnosis (4).

At the same time, suggestibility has been proposed as a potential moderator of treatment response in hypnosis-based interventions. From a personalized or precision medicine perspective, identifying individual characteristics such as suggestibility that may predict differential treatment response is of particular relevance. Empirically, this assumption is supported by meta-analytic findings indicating a small-to-moderate association between suggestibility and clinical outcomes after hypnosis intervention (*r* = 0.24), suggesting that suggestibility accounts for a limited but meaningful proportion of variance in treatment response (5).

Recent systematic reviews further highlight that, despite substantial empirical evidence for the effects of hypnotic suggestions, no consensus exists regarding the mechanisms underlying these effects, with competing theoretical accounts emphasizing different cognitive and social processes (6).

Hypnosis has been investigated as a potential intervention for depressive symptoms.

A meta-analysis of controlled trials reported moderate to large effect sizes for hypnotic interventions in reducing depressive symptoms, with effects comparable to established psychological treatments (7). However, more recent evidence has yielded a more cautious interpretation. A systematic review and meta-analysis of randomized controlled trials concluded that the available evidence remains insufficient and heterogeneous to support firm conclusions regarding the efficacy of hypnosis-based interventions for Major Depressive Disorder across severity levels (8). A rater-blinded randomized controlled trial further reported that hypnotherapy was not inferior to cognitive behavioral therapy in mild to moderate depression (9). Taken together, the current literature suggests that while hypnosis may have clinically relevant effects on depressive symptoms, the strength and consistency of the evidence remain uncertain. However, empirical research directly examining the role of suggestibility in treatment-related changes and in general in depressive symptoms remains limited.

This remains particularly relevant in group-based interventions, where social context and shared expectations may additionally influence responsiveness to suggestions.

Standardized scales are the primary tools for assessing suggestibility in hypnosis research, including instruments such as the Harvard Group Scale of Hypnotic Susceptibility (HGSHS), which captures behavioral responsiveness to suggestions following a hypnotic induction. The Harvard Group Scale of Hypnotic Susceptibility, *Form 5: German version* (HGSHS-5:G), a recently developed short form of the HGSHS based on German normative data (10), includes only motor challenge items (e.g., arm immobility, finger lock, arm rigidity), thereby reducing administration time. Initial studies support its reliability and validity (10). However, systematic evaluations in clinical samples, such as patients with depressive disorders, are still lacking. To complement these behavioral measures within hypnotic contexts, imaginative aspects of suggestibility can be assessed using instruments such as the Creative Imagination Scale (CIS), an induction-free measure that captures responsiveness to suggestions through voluntary imaginative processes (12). Together, these measures allow for a more differentiated assessment of suggestibility by capturing both behavioral and imaginal aspects, which may reflect distinct pathways through which hypnotic interventions exert their effects.

The objective of this exploratory analysis of a pilot randomized controlled trial was to examine whether hypnotic suggestibility, assessed using the Harvard Group Scale of Hypnotic Susceptibility, Form 5: German version (HGSH-5:G), and the CIS, is associated with changes in depressive symptoms over time, and whether this association differs between standardized group hypnosis, progressive muscle relaxation, and a control group in individuals with mild to moderate depressive symptoms.

## 2 Materials and methods

### 2.1 Sample and study context

The present analysis draws on data from the HypnoDeep trial, a prospective, randomized, controlled, open-label, exploratory telemedical pilot trial. The parent trial was designed to explore the feasibility and potential effects of a six-week telemedical group program with hypnotherapy plus routine care compared with progressive muscle relaxation plus routine care and routine care alone in adults with depressive symptoms and elevated stress. The trial was conceived as a pilot study to inform the planning of a future confirmatory randomized trial rather than as a confirmatory efficacy trial. Detailed trial procedures are described separately (DRKS00032115). Participants were recruited via online advertisements, newsletters, and outpatient care networks and completed an initial online screening. All participants provided written informed consent prior to participation.

A total of 100 participants were randomized to one of three study groups: group hypnosis, PMR, or wait-list control. Of these, three participants did not complete baseline assessments and were excluded, resulting in a total trial sample of 97 participants. Baseline questionnaires and sociodemographic information were collected prior to intervention onset.

Participants met ICD-10 (International Statistical Classification of Diseases and Related Health Problems, 10. Revision) criteria for a mild depressive episode or recurrent depression (F32.0 or F33.0) and reported elevated stress levels (≥ 40 mm on a visual analogue scale). Exclusion criteria included psychotic disorders, post-traumatic stress disorder, personality disorders, acute suicidality, substance misuse, and severe medical conditions likely to interfere with study participation. A full list of inclusion and exclusion criteria is provided in Supplementary file 1.

Self-reported depressive symptom severity was assessed using the Beck Depression Inventory–II (BDI-II) at baseline and after 6 weeks. ICD-10 classification was based on a clinical interview conducted at baseline.

### 2.2 Interventions

Participants in the hypnosis and PMR groups attended six weekly 90-min telemedical group sessions (up to 10 participants per group) and were instructed to practice the respective techniques at home for 15–20 min at least three times per week. Detailed descriptions of all session procedures, thematic content, and therapeutic components for both interventions are provided in Supplementary file 2 (PMR) and Supplementary file 3 (hypnosis).

The hypnosis protocol consisted of a standardized induction, deepening phase, and therapeutic suggestions targeting relaxation, affect regulation, and ego-strengthening. All sessions followed standardized scripts; no individual tailoring was applied.

The hypnosis and PMR interventions were implemented as add-on interventions, meaning that participants were not required to discontinue ongoing evidence-based treatments such as psychotherapy or antidepressant medication. Participants in all groups could continue usual care throughout the trial.

Sessions were conducted by trained facilitators with formal certification in clinical hypnosis, all of whom were licensed psychotherapists or board-certified psychiatrists with experience in delivering structured group interventions.

Treatment fidelity was ensured through adherence to standardized scripts and session protocols. Facilitators documented session content and deviations, and the study team monitored adherence through regular supervision.

Due to the nature of the interventions, participants and facilitators were not blinded to group allocation. Outcome assessments were self-reported online, and data analysts were blinded to group labels during statistical modeling.

### 2.3 Measures

Assessments were conducted at baseline, 6 weeks, and 14 weeks across all groups using online questionnaires. The present analyses focused on the six-week assessment, as this time point coincided with the end of the intervention phase.

Hypnotic suggestibility was assessed at baseline using the CIS and the HGSH-5-G, 5-item short form (HGSHS-5:G). All measures were administered online. Scoring followed the official manuals for each instrument.

#### 2.3.1 Harvard group scale of hypnotic susceptibility—5-item short form (HGSHS-5:G)

Suggestibility was assessed using the HGSHS-5:G, a recently validated German short form of the Harvard Group Scale of Hypnotic Susceptibility, Form A (10, 11, 34). The instrument consists of five behavioural motor tasks assessing responsiveness to hypnotic suggestions. Each item is scored dichotomously (0 = not passed, 1 = passed), resulting in a summed score ranging from 0 to 5. The HGSHS-5:G has demonstrated high reliability and validity (10).

#### 2.3.2 Creative imagination scale (CIS)

Suggestibility was also assessed using the Creative Imagination Scale [CIS; (13)], a self-report instrument comprising 10 items that can be administered without a formal hypnotic induction. The CIS assesses a variety of imaginative responses, including motor and sensory experiences as well as alterations in perception and time experience. Participants rated the degree to which each suggested experience matches their own subjective experience on a five-point scale ranging from 0 (“not at all”) to 4 (“almost exactly”), yielding a total score between 0 and 40. The present study employed a German-language version of the CIS (14), which was evaluated psychometrically for the first time in this sample in another publication. The CIS has also shown evidence of reliability and validity, with normative data available (12).

### 2.4 Statistical analyses

The parent HypnoDeep trial was designed as a prospective, randomized, controlled, open-label, exploratory telemedical pilot trial and was not intended for confirmatory hypothesis testing. Accordingly, no formal *a priori* sample size calculation was performed, and the findings of both the parent trial and the present secondary moderator analysis were interpreted as exploratory. Analyses were conducted in R [version 4.5.1; (15)].

Baseline sociodemographic and clinical characteristics were summarized for the full study sample and stratified by randomized group using means, standard deviations, medians, and quartiles for continuous variables and absolute and relative frequencies for categorical variables. In addition, baseline characteristics were compared descriptively between participants with complete outcome data and those with missing follow-up data. Further, baseline characteristics were also summarized in strata defined by median splits of the HGSHS-5:G and CIS scores.

To examine whether hypnotic suggestibility was associated with change in depressive symptoms over time and whether such associations differed between treatment groups, linear mixed-effects models were fitted with BDI-II scores as the dependent variable. This modelling approach was chosen because depressive symptoms were assessed repeatedly within participants at baseline and after 6 weeks. Fixed effects included time (baseline vs. 6 weeks), treatment group, baseline suggestibility, all corresponding two-way interactions, and the three-way interaction between time, suggestibility, and treatment group. Separate models were estimated for the suggestibility measures and both were z-standardized prior to analysis. Age was entered as a mean-centered covariate, and sex was included as an additional covariate. A random intercept for participants was specified to account for within-subject correlation due to repeated measurements. Models were estimated using restricted maximum likelihood (REML).

The primary parameter of interest was the interaction between time, suggestibility, and treatment group, which tested whether baseline suggestibility differentially moderated change in depressive symptoms across the hypnosis, PMR, and control groups. Suggestibility was modeled continuously throughout; no dichotomization was used in the inferential analyses.

All participants with available baseline BDI-II data and non-missing baseline HGSHS-5:G or CIS values were included in the primary analyses, allowing missing follow-up observations under a missing-at-random assumption. As a sensitivity analysis, missing BDI-II values at 6 weeks were multiply imputed using predictive mean matching. Imputation models included treatment group, age, sex, baseline suggestibility, and baseline BDI-II. The mixed-effects models were then re-estimated in each imputed dataset (*m* = 20) and pooled using Rubin’s rules.

Model fit was summarized using the Akaike information criterion (AIC), Bayesian information criterion (BIC), log-likelihood, and marginal and conditional *R*^2^. Model assumptions were evaluated by inspection of residual distributions and homoscedasticity. Multicollinearity among fixed effects was assessed, and the intraclass correlation coefficient (ICC) was calculated to quantify within-subject clustering. Fixed-effect estimates are reported with regression coefficients, standard errors, t-values, *p*-values, and 95% confidence intervals. Influence diagnostics were conducted at the participant level using Cook’s distance and DFBETAs based on case-deletion methods (threshold: Cook’s distance > 4/n).

For visualization, descriptive BDI-II values were plotted separately for low and high levels of baseline suggestibility, based on a median split, across treatment groups and time points for both suggestibility measures. In addition, distributions of baseline HGSHS-5:G and CIS scores and scatterplots illustrating the associations between baseline suggestibility and change in BDI-II scores from baseline to 6 weeks were examined descriptively.

To assess the sensitivity of the study to detect effect sizes reported in prior literature, a *post hoc* power analysis was conducted. Statistical power was calculated for correlation effect sizes (*r* = 0.15, 0.24, 0.30, and 0.50), assuming a two-tailed alpha level of 0.05 and the complete-case sample size within individual treatment groups (e.g., *N* = 24 in the hypnosis group). Because prior meta-analytic studies have reported associations between hypnotic suggestibility and treatment outcomes as correlations, these effect sizes were used as an approximate benchmark for the detectable effect sizes in the present study.

## 3 Results

### 3.1 Study sample

The full study sample comprised 97 participants. For the HGSHS-5:G analyses, 94 participants contributed data to the mixed-effects model (32 in the control group, 32 in the hypnosis group, and 30 in the PMR group). For the CIS analyses, 94 participants contributed data (32 in the control group, 33 in the hypnosis group, and 29 in the PMR group). The complete-case sample with observed suggestibility score (HGSHS-5:G or CIS) and follow-up outcome (BDI-II) included 78 participants (24 in hypnosis, 24 in PMR, and 30 in control).

Baseline characteristics by study group are presented in Table 1. Participants with complete outcome data and those with missing follow-up data were compared descriptively to assess possible attrition bias (Supplementary Table S1). Overall, differences were small, with some variation in sex distribution.

**Table 1:** Baseline characteristics of the randomized participants with available baseline data, overall and by treatment group.

Characteristic	Overall (*n* = 97)	Hypnosis (*n* = 34)	Control (*n* = 32)	PMR (*n* = 31)
Age (years)				
Mean (SD)	39.2 (12.0)	40.3 (11.8)	38.1 (12.1)	39.2 (12.4)
Median (Q1, Q3)	38.0 (29.0, 49.0)	38.5 (32.0, 52.0)	37.5 (29.0, 45.5)	37.0 (29.0, 48.0)
Sex, *n* (%)				
Female	69 (71.1%)	20 (58.8%)	27 (84.4%)	22 (71.0%)
Male	26 (26.8%)	13 (38.2%)	5 (15.6%)	8 (25.8%)
Diverse	2 (2.1%)	1 (2.9%)	0 (0.0%)	1 (3.2%)
Body mass index (kg/m^2^)				
Mean (SD)	22.8 (3.5)	23.3 (3.8)	22.2 (2.3)	22.6 (4.1)
Median (Q1, Q3)	22.0 (20.5, 24.6)	23.2 (20.9, 25.5)	22.0 (21.2, 23.5)	21.1 (19.8, 25.2)
BDI-II score				
Mean (SD)	21.5 (8.1)	23.5 (7.2)	21.8 (9.2)	19.1 (7.3)
Median (Q1, Q3)	21.0 (16.0, 26.0)	21.5 (17.0, 30.0)	20.0 (15.5, 26.5)	20.0 (13.0, 24.0)
HGSHS-5:G score				
Mean (SD)	2.9 (1.6)	3.0 (1.6)	2.5 (1.6)	3.1 (1.7)
Median (Q1, Q3)	3.0 (1.0, 4.0)	3.5 (2.0, 4.0)	3.0 (1.0, 4.0)	3.0 (2.0, 4.5)
CIS score				
Mean (SD)	17.6 (7.7)	17.0 (6.7)	18.6 (8.8)	17.3 (7.5)
Median (Q1, Q3)	18.5 (12.0, 22.0)	16.0 (12.0, 21.0)	19.5 (12.5, 26.0)	19.0 (12.0, 23.0)

BDI-II, beck depression inventory–II; CIS, creative imagination scale; HGSHS-5:G, Harvard group scale of hypnotic susceptibility, 5-item German short form; PMR, progressive muscle relaxation. Higher HGSHS-5:G and CIS scores indicate higher hypnotic suggestibility.

Baseline characteristics stratified by median splits of the CIS and HGSHS-5:G scores are presented in Supplementary Tables S2, S3. Groups were largely comparable, although baseline depressive symptom severity tended to be higher in the low suggestibility groups.

### 3.2 Association between hypnotic suggestibility and change in depressive symptoms

**Figure 1:**
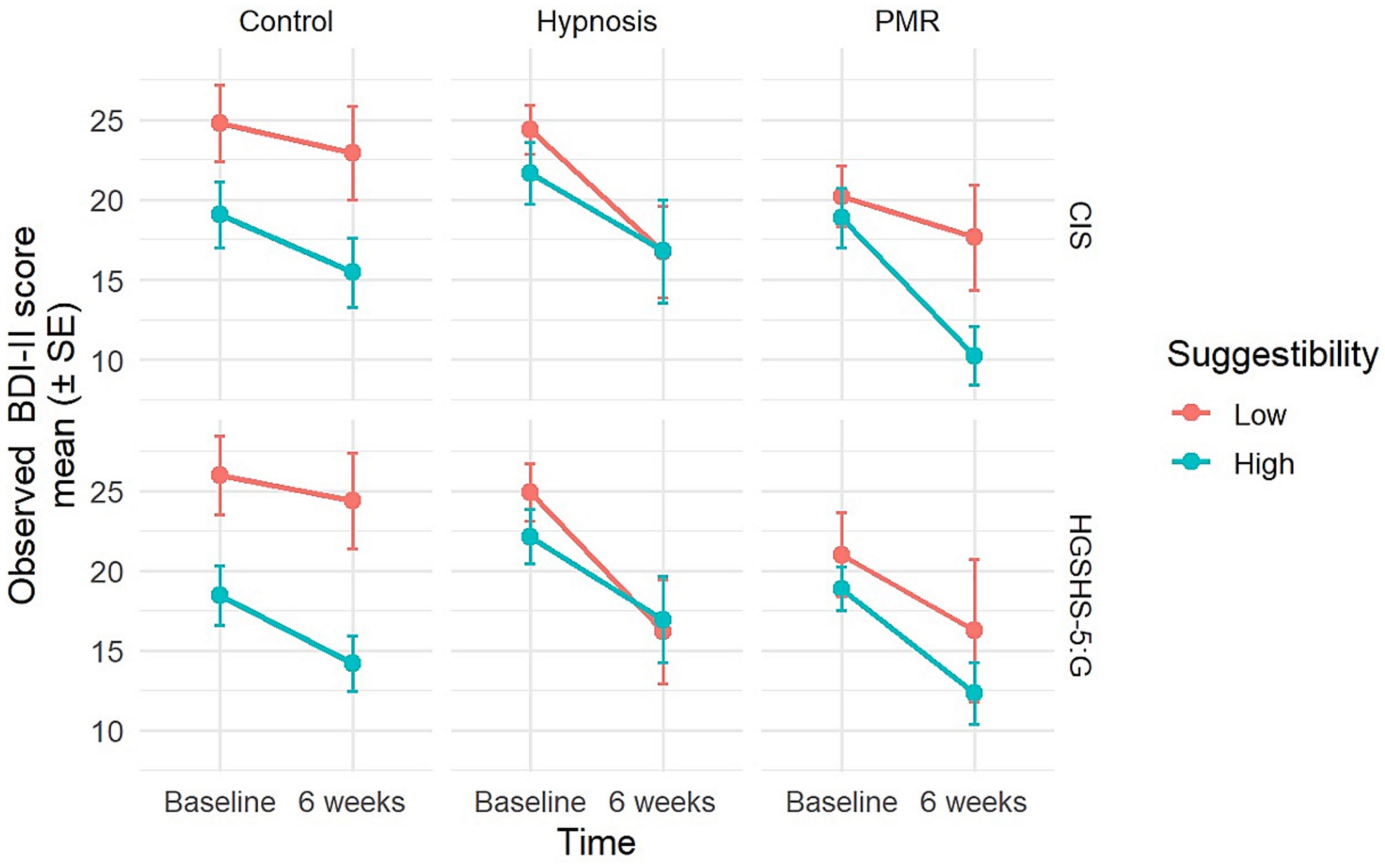
Descriptive mean BDI-II scores (±SE) at baseline and 6 weeks for participants with low and high hypnotic suggestibility, based on a median split of baseline HGSHS-5:G and CIS scores.

Descriptive BDI-II values stratified by low and high levels of baseline suggestibility (median split) across treatment groups and time points are shown in Figure 1. Across groups, depressive symptoms decreased over time, with largely similar patterns for low and high suggestibility. Distributions of suggestibility scores and scatterplots of baseline suggestibility versus change in BDI-II are provided in Supplementary Figure S1.

Descriptive correlation analyses between baseline suggestibility and change in depressive symptoms are presented in Supplementary Table S4. Across the full sample, correlations were small. Correlations within treatment groups were likewise small (range −0.34 to 0.24).

A *post hoc* power analysis based on the complete-case sample indicated that the study was substantially underpowered to detect realistic effect sizes. At a two-tailed alpha level of 0.05, achieved power was 10.8% for a small effect (*r* = 0.15), 20.7% for an effect of *r* = 0.24, 30.3% for a moderate effect (*r* = 0.30), and 72.6% for a large effect (*r* = 0.50). The sample sizes required to achieve 80% power were *N* = 346 for *r* = 0.15, *N* = 134 for *r* = 0.24, *N* = 85 for *r* = 0.30, and *N* = 29 for *r* = 0.50.

#### 3.2.1 Harvard group scale of hypnotic susceptibility—5-item short form

In the regression analysis for HGSHS-5:G, there was no indication that baseline HGSHS-5:G was associated with differential change in depressive symptoms over time in the control group (time × HGSHS-5:G: *β* = −1.20, 95% CI −4.00 to 1.60, *p* = 0.404) (Supplementary Table S5).

The three-way interaction terms likewise did not indicate differential moderation for either hypnosis (*β* = 2.05, 95% CI −2.21 to 6.31, *p* = 0.350) or PMR (*β* = −0.97, 95% CI −5.14 to 3.20, *p* = 0.651), relative to the control group.

Across the model, BDI-II scores were lower at 6 weeks than at baseline (time: *β* = −3.40, 95% CI −6.33 to −0.54, *p* = 0.023). Full model results, model fit indices, intraclass correlation coefficients, and diagnostic results are reported in Supplementary Tables S5, S6. Influence diagnostics across models did not indicate substantial influence of individual cases, supporting the robustness of the model estimates.

Results of the multiple-imputation sensitivity analysis were closely aligned with the primary analysis. No evidence was found for a time × HGSHS-5:G interaction (*β* = −1.14, 95% CI −3.95 to 1.66, *p* = 0.423), and the three-way interaction terms remained compatible with no differential moderation by treatment group.

#### 3.2.2 Creative imagination scale

In the regression analysis for CIS, there was no indication that baseline CIS was associated with differential change in depressive symptoms over time in the control group (time × CIS: *β* = −0.94, 95% CI −3.35 to 1.46, *p* = 0.444) (Supplementary Table S7).

The three-way interaction terms did not indicate differential moderation for hypnosis (*β* = 3.06, 95% CI −1.36 to 7.48, *p* = 0.179) or PMR (*β* = −0.44, 95% CI −4.49 to 3.61, *p* = 0.833), relative to the control group.

BDI-II scores were again lower at 6 weeks than at baseline (time: *β* = −2.96, 95% CI −5.75 to −0.17, *p* = 0.041). Full model results, model fit indices, intraclass correlation coefficients, and diagnostic results are reported in Supplementary Tables S7, S8. Influence diagnostics across models did not indicate substantial influence of individual cases, supporting the robustness of the model estimates.

Findings from the multiple-imputation sensitivity analysis were consistent with the main analysis. No evidence was observed for a time × CIS interaction (*β* = −0.97, 95% CI −3.44 to 1.50, *p* = 0.439), and the three-way interaction terms remained compatible with no differential moderation by treatment group.

## 4 Discussion

The present exploratory analysis examined whether there is any indication that baseline hypnotic suggestibility may be associated with changes in depressive symptoms over time and whether such associations differ between hypnosis, PMR, and the control group. Across both suggestibility measures (HGSHS-5:G and CIS), no clear evidence was observed that suggestibility was related to differential change in depressive symptoms. Likewise, there was no indication that the association between suggestibility and symptom change differed between groups.

Within the limits of this exploratory design, these findings do not provide support for the assumption that suggestibility plays a differential or hypnosis-specific role in symptom change. However, this interpretation must be considered with caution in light of the limited statistical power of the study. Prior meta-analytic findings suggest small-to-moderate associations between suggestibility and treatment outcomes [e.g., *r* = 0.24; (5)]. Given the present sample size, the study was not sufficiently powered to reliably detect effects of this magnitude.

### 4.1 Strengths and limitations

Strengths of this randomized controlled clinical study include the use of two standardized questionnaires of hypnotic suggestibility, a longitudinal design with repeated symptom assessment, and the application of mixed-effects models to account for individual variability. In addition, the use of two conceptually distinct suggestibility measures allowed for a broader assessment of the construct across behavioral and imaginative domains.

However, several limitations warrant consideration. First, an important limitation of this exploratory study is its limited statistical power. The *post hoc* power analysis demonstrated that the complete-case sample sizes within the treatment groups were underpowered to detect realistic small-to-moderate effect sizes. Accordingly, the absence of a statistically significant association between hypnotic suggestibility and symptom change cannot be interpreted as evidence for the absence of an effect, but may instead reflect insufficient statistical power. Furthermore, as the primary analyses were based on interaction effects in a repeated-measures mixed-effects model, statistical power to detect such effects may be even lower than suggested by these correlation-based estimates.

Second, causal inferences regarding the moderating role of hypnotic suggestibility are not warranted, and any observed associations may reflect general or nonspecific treatment effects rather than mechanisms specific to hypnosis. Third, generalisability of the findings is limited. The sample was restricted to individuals with mild to moderate depressive symptoms and elevated stress levels, which may not extend to more severe or clinically complex populations. In addition, the intervention was delivered in a standardized telemedical group format, which differs from individualized or face-to-face hypnosis settings and may influence both treatment response and the role of suggestibility. Furthermore, the specific intervention protocol used in this trial may not generalize to other hypnosis approaches, limiting the transferability of the findings across therapeutic formats and procedures. Fourth, hypnotic suggestibility was assessed using two conceptually distinct instruments with inherent limitations, capturing different aspects of the construct. Fifth, no manipulation check (e.g., subjective hypnotic depth or engagement) was included. As suggestibility was assessed only at baseline, it remains unclear whether hypnotic processes were consistently and effectively induced during the intervention, which may have obscured potential moderation effects. Sixth, the possibility of bias due to differential attrition cannot be fully excluded. To address this, completers and non-completers were compared across baseline variables, including suggestibility, depressive symptoms, age, sex, and BMI. Nevertheless, the absence of statistically significant differences does not preclude subtle systematic dropout, and any such bias may have influenced the observed associations and reduced the precision of the estimates. Taken together, these limitations indicate that the present findings should be interpreted as preliminary and hypothesis-generating rather than definitive evidence regarding the role of hypnotic suggestibility in treatment outcomes.

In addition, the present moderator analysis extends the original exploratory aims of the pilot trial and should therefore be regarded as hypothesis-generating. Although suggestibility was assessed at baseline as part of the parent trial, the study was not designed to provide confirmatory evidence for suggestibility as a moderator of treatment response.

### 4.2 Interpretation of findings

Although hypnotic suggestibility has traditionally been conceptualized as a facilitator of responsiveness to hypnotic interventions, empirical evidence for this assumption has been mixed. Meta-analytic findings indicate that suggestibility accounts for only a modest proportion of variance in clinical outcomes (approximately 6% in clinical settings) (5). In line with this, recent umbrella reviews highlight substantial variability in the efficacy of hypnosis across clinical domains and emphasize the need for further research on moderators of treatment response (16). Moreover, narrative reviews and methodological critiques point to heterogeneous findings across conditions and study designs, as well as limitations in the measurement of hypnotic suggestibility that may contribute to inconsistent results (17, 18). The present findings add to this literature by suggesting that, within this sample of individuals with mild to moderate depressive symptoms receiving standardized group hypnosis, higher suggestibility was not clearly associated with greater symptom reduction.

Several explanations may account for this result. First, hypnotic suggestibility as measured by behavioural or imaginative scales may not adequately capture the cognitive processes most relevant for therapeutic change in depression, such as expectancy formation or broader top-down regulatory mechanisms. In addition, processes such as alterations in the sense of agency and the capacity for phenomenological control may be relevant for understanding how suggested experiences are subjectively experienced as real. Contemporary integrative models emphasize that hypnotic responding reflects dynamically interacting cognitive, social, and neurophysiological processes, including response expectancies and cognitive engagement (4). Complementarily, hypnosis has been conceptualized as a form of top-down regulation of consciousness in which verbal suggestions can elicit pronounced changes in the contents of consciousness and a wide range of psychological functions (19). Second, although suggestibility was assessed at baseline, the use of standardized scripts and a uniform delivery format in group hypnosis may constrain the extent to which individual differences in hypnotic suggestibility can be leveraged during treatment, potentially attenuating their influence on symptom change compared to individualized hypnotic interventions. Third, the study may have been underpowered to detect small-to-moderate moderation effects. Fourth, the present analyses assumed linear relationships between suggestibility and treatment response; potential nonlinear or threshold effects could therefore not be captured. Fifth, hypnotic suggestibility does not represent a unitary construct, and different standardized measures appear to index partially distinct abilities rather than a single trait.

### 4.3 Alignment with previous evidence

The present findings are broadly in line with prior studies suggesting that hypnotic suggestibility may not be a necessary or sufficient condition for clinical improvement following hypnosis based interventions (5). Whereas some prior studies have reported modest associations between suggestibility and treatment outcome [e.g., (5)], narrative reviews and theoretical accounts have highlighted the heterogeneity and inconsistency of these findings (18, 20). The current exploratory study examined suggestibility in a routine clinical context using a standardized group intervention and a sample characterized by mild depressive symptoms. Variability in previous findings may partly reflect heterogeneity in intervention formats, outcome domains, and the operationalisation of hypnotic suggestibility, thereby limiting the comparability of results across studies.

Beyond the hypnosis literature, suggestibility has also been implicated in treatment response across non-hypnotic therapeutic contexts. For example, higher baseline suggestibility has been associated with early symptom changes and side-effect reporting during antidepressant pharmacotherapy, presumably by shaping expectations rather than reflecting a treatment-specific mechanism (21). This aligns with broader theoretical perspectives suggesting that suggestibility may index general responsiveness to contextual, expectancy-driven, or interpersonal processes rather than mechanisms unique to hypnosis.

Experimental pain research further indicates that suggestibility can moderate hypnotic analgesia, but these effects appear to depend strongly on context, measurement range, and intervention characteristics. Milling et al. (22) demonstrated that suggestibility predicted pain reduction specifically in a hypnotic intervention but not in a cognitive-behavioral analogue, while response expectancies and treatment credibility independently mediated outcomes across both modalities. These findings suggest that the influence of suggestibility is context-dependent and may vary according to intervention characteristics and outcome domains.

By assessing both behavioural (HGSHS-5:G) and imaginative (CIS) measures of hypnotic suggestibility within a longitudinal clinical trial framework, the present study extends existing work by providing convergent evidence of null associations across different operationalisations of suggestibility. Taken together, these findings add to the mixed evidence regarding the role of hypnotic suggestibility in treatment response and underscore the importance of considering intervention format, sample characteristics, and outcome context when evaluating its clinical relevance.

Beyond individual clinical decision-making, broader considerations also argue against routine screening for hypnotic suggestibility. From a policy and cost-effectiveness perspective, implementing suggestibility assessments in routine care would require additional resources without clear evidence of clinical benefit. Moreover, such screening could inadvertently introduce inequities, as suggestibility measures may be influenced by cultural, linguistic, or cognitive factors that are not directly related to treatment responsiveness. Given these concerns, and in line with prior meta-analytic conclusions questioning the clinical utility of suggestibility assessment (5), routine screening does not appear justified from a clinical, ethical, or health-system perspective.

### 4.4 Heterogeneity and implications for mechanism research

Although the present study did not provide evidence that hypnotic suggestibility moderated treatment response, the findings highlight the continued need to investigate factors that may contribute to variability in outcomes following hypnosis-based interventions. These findings align with broader meta-analytic evidence demonstrating substantial variability in the efficacy of hypnosis across clinical domains and highlighting the need for research on moderators of treatment response (16). In parallel, neurophysiological and neuroimaging research indicates that the mechanisms underlying hypnotic interventions remain incompletely understood and highly heterogeneous. Reviews and experimental studies highlight distributed, context-dependent neural processes rather than a single neurobiological signature of hypnosis, underscoring the exploratory state of mechanism research, including in depression (19, 23–26).

Several psychological mechanisms have been proposed to explain why suggestibility might influence treatment outcomes. These include stronger expectations about improvement, greater openness and engagement with therapeutic procedures, and a higher capacity for focused attention and imaginative involvement. Such processes could, in principle, amplify the impact of therapeutic suggestions across different treatment contexts.

Potential candidates include treatment expectations and credibility-related attitudes toward hypnosis. However, direct associations between baseline outcome expectations and hypnotic intervention outcomes have been inconsistent, with some studies finding no significant effects of outcome expectations on symptom change (28). This underscores the importance of further investigating process variables in hypnosis-based treatments.

In addition, broader psychotherapy research has identified the therapeutic alliance as a process variable that is robustly and consistently associated with treatment outcomes, including symptom change in depression and other conditions (28, 29).

Research on moderators such as baseline clinical features (e.g., number of prior episodes) indicates that client characteristics can influence process–outcome associations in psychotherapy for depression (30). Hypnotic treatment effects appear to reflect a multicomponent process involving interacting biological, psychological, and social factors, rather than a single underlying mechanism (31). Neurocognitive studies indicate that hypnotic responding is associated with heterogeneous neural dynamics, including large-scale network connectivity changes during induction, localized effects of suggestion, and modulation by individual susceptibility (32). These findings suggest that individual differences constitute plausible predictors of variability in hypnosis-based treatment outcomes. Beyond quantitative approaches, future research could also incorporate qualitative methodologies to explore additional factors that may contribute to differential treatment response.

## 5 Conclusion

In summary, the present analyses did not provide indications that hypnotic suggestibility moderates changes in depressive symptoms following standardized group hypnosis or PMR. Given the exploratory design and limited statistical power, causal inferences regarding the moderating role of hypnotic suggestibility cannot be drawn, and the findings should be interpreted as preliminary.

Future studies with larger samples are needed to clarify the role of suggestibility and to examine additional psychological and contextual factors that may contribute to individual differences in response to hypnosis-based interventions.

## Data Availability

The raw data supporting the conclusions of this article will be made available by the authors, without undue reservation.
