# Supplementary material for: Hypnotic suggestibility as a moderator of treatment response in mild to moderate depression: an exploratory secondary analysis
Source: Front Med (Lausanne). 2026 Jul 2;13:1847384. doi: 10.3389/fmed.2026.1847384 (PMC13373041; doi:10.3389/fmed.2026.1847384)
Supplement: Supplementary file 1 [file Supplementary_file_1.DOCX]

**Supplementary Material 1.** Inclusion and Exclusion Criteria

**Inclusion Criteria**

- Age 18–70 years
- Diagnosis of mild depressive episode or recurrent mild depression (ICD‑10: F32.0 or F33.0)
- Self‑reported stress level ≥ 40 mm on a 100‑mm visual analogue scale during the preceding week
- Sufficient German language proficiency
- Technical ability to participate in telemedical group sessions and complete online questionnaires
- Capacity to provide written informed consent

**Exclusion Criteria**

- Current or past psychotic disorder
- Post‑traumatic stress disorder
- Personality disorder
- • Moderate or severe depressive episode according to ICD‑10 (F32.1, F32.2, F33.1, F33.2)
- Severe obsessive‑compulsive disorder
- Acute suicidality or recent self‑harm
- Alcohol or drug dependence
- Severe acute or chronic medical conditions interfering with participation
- Participation in another depression study within the previous two months
- Ongoing or recent (past two months) use of hypnosis, self‑hypnosis, autogenic training, or progressive muscle relaxation
- Insufficient German language proficiency
- Ongoing early retirement procedure
