# Supplementary material for: Hypnotic suggestibility as a moderator of treatment response in mild to moderate depression: an exploratory secondary analysis
Source: Front Med (Lausanne). 2026 Jul 2;13:1847384. doi: 10.3389/fmed.2026.1847384 (PMC13373041; doi:10.3389/fmed.2026.1847384)
Supplement: Supplementary file 2 [file Supplementary_file_2.DOCX]

**Supplementary Material 2.** Description of the Progressive Muscle Relaxation (PMR) study intervention. This table details the thematic focus and procedural components of each of the six weekly online sessions (approximately 90 minutes per session), delivered in groups of up to 10 participants.

| Themes | Procedures | Duration  (minutes) |
| --- | --- | --- |
| **1. Session**  Relaxation of hands and arms | 1. Getting acquainted  2. Information about PMR  3. Demonstration of the muscle groups  4. Practical exercise part  5. Debriefing  6. Homework: Initiate daily exercising of PMR recordings and closing | 20  20  5  15  30 |
| **2. Session**  Relaxation of feet, calves, tighs and buttocks is added | 1. Exploration of the experiences with practicing independently at home  2. Psychoeducation: Stress and Depression  3. Demonstration of the muscle groups  4. Practical exercise part  5. Debriefing  6. Homework: Exercise daily of the PMR recordings and closing | 5  10  10  25  30 |
| **3. Session**  Relaxation of forehead, eyes, jaw and neck is added | 1. Exploration of the experiences with practicing independently at home  2. Psychoeducation: Stressors  3. Demonstration of the muscle groups  4. Practical exercise part  5. Debriefing  6. Homework: Exercise daily of the PMR recordings and closing | 5  10  10  25  30 |
| **4. Session**  Relaxation of shoulder, breast, back and abs is added | 1. Exploration of the experiences with practicing independently at home  2. Demonstration of the muscle groups  3. Practical exercise part  4. Debriefing  5. Homework: Exercise daily of the PMR recordings and closing | 15  5  40  30 |
| **5. Session**  Repetition of fourth session | 1. Exploration of the experiences with practicing independently at home  2. Practical exercise part  3. Debriefing  4. Homework: Exercise daily of the PMR recordings and closing | 15  40  30 |
| **6. Session**  Short version | 1. Exploration of the experiences with practicing independently at home  2. Demonstration of the muscle groups of the short version  3. Practical exercise part  4. Debriefing  5. Closing | 15  10  10  15  15  20 |
