# Supplementary material for: Hypnotic suggestibility as a moderator of treatment response in mild to moderate depression: an exploratory secondary analysis
Source: Front Med (Lausanne). 2026 Jul 2;13:1847384. doi: 10.3389/fmed.2026.1847384 (PMC13373041; doi:10.3389/fmed.2026.1847384)
Supplement: Supplementary file 3 [file Supplementary_file_3.DOCX]

**Supplementary Material 3.** Description of the Hypnotherapy study intervention. This table details the thematic focus and procedural components of each of the six weekly online sessions (approximately 90 minutes per session), delivered in groups of up to 10 participants.

| Themes | Procedures | Duration  (minutes) |
| --- | --- | --- |
| **1. Session**  Information about stress and first experience with hypnosis | 1. Getting acquainted, promotion of group cohesion and rapport  2. Psychoeducation: Stress and stress management  3.“Lemon Exercise” as a convincer  4. Hypnosis: First experience with hypnosis for physiological relaxation and mental calming  a. Induction: Breathing and contact with the surface  b. Distancing technique: With each exhale, place thoughts/issues into the balloon, let go of the band and let the balloon fly  c. Deepening: Focusing attention on the breath  d. Mental reassurance: Imagining the mind as a pond according to Stanton,^63^ positive suggestions for calm and relaxation  e. Anchoring: Hand on a comfortable, relaxed part of the body  f. Psychoeducation: For the stress and relaxation reaction in trance to promote the natural autonomic self-regulation  g. Post-hypnotic suggestion: Practice independently and let the positive experience take effect  h. Reorientation: 3-2-1: Arms - breath – eyes  5. Debriefing  6. Homework: Initiate daily exercising of hypnosis recordings and closing | 10  25  10  25  20 |
| **2. Session**  Finding a specific resource for specific situation & resource activation in hypnosis | 1. Exploration of the experiences with practicing independently at home 2. Imparting the construct “resource” 3. Idendification of necessary resources for the specific stressful situations of the participants  4. Hypnosis: Distancing technique and resourceful place  a. Seeding  b. Induction: Eye fixation  c. Deepening and distancing technique: Breathing, bridge with suitcase in which stressful topics can be put in  d. At the end of the bridge, resource activation at the resourceful place with possibilities: specific experienced situation in which the resource can be experienced, a specific place; a color impression; deepened emotional and physical experience;  e. Anchoring: Finger closure  f. Post-hypnotic suggestion: Return to this place at any time, should something appealing be taken with from this place  g. Return: Bridge back; suitcase: decision as to whether something should be taken;  h. Reorientation: 3-2-1: Arms - Breathing – Eyes  5. Debriefing  6. Homework: Exercise daily of hypnosis recordings and closing | 10  20  40  20 |
| **3. Session**  Deepening resource activation in specific situation | 1. Exploration of the experiences with practicing independently at home  2. Hypnosis: Extension and deepening  a. Seeding  b. Induction: Eye fixation  c. Deepening and distancing technique: Breathing; bridge with suitcase  d. At the end of the bridge, resource activation at the resourceful place with possibilities: specific experienced situation in which the resource can be experienced; a specific place; a color impression; deepened emotional and physical experience;  e. Anchoring: Finger closure  f. Suggestion of helpful attitudes towards stress management and performance orientation according to Stanton^64^  g. Reorientation: 3-2-1: Arms - Breathing – Eyes  3. Debriefing  4. Homework: Exercise daily of hypnosis recordings and closing | 15  50  25 |
| **4. Session**  Training a quick and easy resource activation via stress anchor (= resource key) | 1. Exploration of the experiences with practicing independently at home  2. Teaching the “resource key” technique  3. Hypnosis: Repetition of session three “resource activation” with possibility of additional anchors for the resource experience: resource symbol, resource word, sound, color  4. Hypnosis: Practicing the rapid occurrence of resource experience  5. Identifying the first signals for the occurrence of a stress reaction with subsequent resourceful place  6. Hypnosis: Practicing the reflexive triggering of the resource experience through a stress stimulus (“resource key”) according to Bongartz’ “problem as anchor”^65^  7. Debriefing  8. Homework: Exercise daily of hypnosis recordings and closing | 10  10  25  10  15  20 |
| **5. Session**  Resource transfer: Future progression in the demanding situation | 1. Exploration of the experiences with practicing independently at home  2. Hypnosis: Resource Transfer  a. Seeding  b. Induction: Eye fixation  c. Deepening and distancing technique: Focusing attention on body and breath, pleasant feeling, bridge and suitcase  d. Resource activation: Resourceful place  e. Resource transfer: Future progression from resourceful place to the demanding situation; first cue of an incipient stress reaction (situative, cognitive, vegetative) as a sign to activate resource experience (applying the “resource key”)  f. Positive suggestions for self-efficacy  g. Positive suggestions for self-confidence  h. Posthypnotic suggestion for success and competence in future situations  i. Reorientation: 3-2-1: Arms - Breathing – Eyes  3. Debriefing  4. Homework: Bring a little stone, exercise daily of hypnosis recordings and closing | 15  50  25 |
| **6. Session**  Future progression with better stress management skills as an integrated part of self-image | 1. Appreciation of the changes in stress coping competences achieved so far and integration into self-image  2. Hypnosis: Marble technique  a. Introduction: Pick up the stone  b. Induction: Focusing attention on the stone  c. Deepening: Relaxation suggestion  d. Partial age regression into the last few weeks of the stress management program: visualization of the trained skills (bridge, resource key, resourceful place, symbol), the improved stress management skills, the changed experience and self-image  e. Future progression to a time in the future when improved stress management skills have become even more entrenched and an integral part of self-image  f. Positive suggestions for self-confidence  g. Anchoring the most important experiences with the stone in the hand  h. Reorientation: 3-2-1: Arms - Breathing – Eyes  3. Debriefing  4. Closing | 25  40  25 |
