# Supplementary material for: Hypnotic suggestibility as a moderator of treatment response in mild to moderate depression: an exploratory secondary analysis
Source: Front Med (Lausanne). 2026 Jul 2;13:1847384. doi: 10.3389/fmed.2026.1847384 (PMC13373041; doi:10.3389/fmed.2026.1847384)
Supplement: Supplementary file 4 [file Table_1.docx]

**Abbreviations:** BDI-II, Beck Depression Inventory–II; CIS, Creative Imagination Scale; HGSHS-5:G, Harvard Group Scale of Hypnotic Susceptibility, 5-item German short form; PMR, progressive muscle relaxation; SD, standard deviation; SE, standard error; CI, confidence interval; ICC, intraclass correlation coefficient; AIC, Akaike information criterion; BIC, Bayesian information criterion.

**Notes Tables S1 – S3:** Continuous variables are presented as mean (SD) and median (Q1, Q3); categorical variables as n (%). P-values are based on group comparisons (t-tests or chi-square tests as appropriate). Effect sizes are reported as Cohen’s d for continuous variables and Cramér’s V for categorical variables.

Table S1: Baseline characteristics of the study sample stratified by endpoint availability (complete cases vs. dropouts).

| **Characteristic** | **Overall** **(n = 97)** | **Complete cases**  **(n = 78)** | **Dropouts** **(n = 19)** | **p** | **Effect size** |
| --- | --- | --- | --- | --- | --- |
| **Age (years)** |  |  |  | 0.114 | d = 0.36 |
| *Mean (SD)* | 39.2 (12.0) | 40.1 (12.4) | 35.7 (9.9) |  |  |
| *Median (Q1, Q3)* | 38.0 (29.0, 49.0) | 38.0 (30.0, 52.0) | 37.0 (26.0, 46.0) |  |  |
| **Sex, n (%)** |  |  |  | 0.034 | V = 0.22 |
| *Female* | 69 (71.1%) | 60 (76.9%) | 9 (47.4%) |  |  |
| *Male* | 26 (26.8%) | 17 (21.8%) | 9 (47.4%) |  |  |
| *Diverse* | 2 (2.1%) | 1 (1.3%) | 1 (5.3%) |  |  |
| **Body mass index (kg/m²)** |  |  |  | 0.083 | d = -0.58 |
| *Mean (SD)* | 22.8 (3.5) | 22.4 (3.1) | 24.4 (4.5) |  |  |
| *Median (Q1, Q3)* | 22.0 (20.5, 24.6) | 22.0 (20.2, 24.1) | 22.7 (20.8, 29.1) |  |  |
| **BDI-II score** |  |  |  | 0.776 | d = -0.08 |
| *Mean (SD)* | 21.5 (8.1) | 21.4 (8.0) | 22.0 (8.4) |  |  |
| *Median (Q1, Q3)* | 21.0 (16.0, 26.0) | 20.0 (16.0, 26.0) | 21.0 (15.0, 27.0) |  |  |
| **HGSHS-5:G score** |  |  |  | 0.486 | d = 0.19 |
| *Mean (SD)* | 2.9 (1.6) | 2.9 (1.7) | 2.6 (1.6) |  |  |
| *Median (Q1, Q3)* | 3.0 (1.0, 4.0) | 3.0 (1.0, 4.0) | 3.0 (1.5, 4.0) |  |  |
| **CIS score** |  |  |  | 0.994 | d = 0.00 |
| *Mean (SD)* | 17.6 (7.7) | 17.6 (7.7) | 17.6 (7.7) |  |  |
| *Median (Q1, Q3)* | 18.5 (12.0, 22.0) | 19.0 (12.0, 22.0) | 16.0 (12.0, 25.5) |  |  |

Table S2: Baseline characteristics of the study sample stratified by suggestibility (CIS median split).

| **Characteristic** | **Overall (n = 94)** | **Low (n = 47)** | **High (n = 47)** | **p** | **Effect size** |
| --- | --- | --- | --- | --- | --- |
| **Age (years)** |  |  |  | 0.233 | d = -0.25 |
| *Mean (SD)* | 39.3 (12.1) | 37.8 (11.5) | 40.8 (12.7) |  |  |
| *Median (Q1, Q3)* | 38.0 (29.0, 50.0) | 37.0 (29.0, 48.0) | 40.0 (29.0, 53.0) |  |  |
| **Sex, n (%)** |  |  |  | 0.206 | V = 0.11 |
| *Female* | 66 (70.2%) | 30 (63.8%) | 36 (76.6%) |  |  |
| *Male* | 26 (27.7%) | 15 (31.9%) | 11 (23.4%) |  |  |
| *Diverse* | 2 (2.1%) | 2 (4.3%) | 0 (0.0%) |  |  |
| **Body mass index (kg/m²)** |  |  |  | 0.332 | d = -0.20 |
| *Mean (SD)* | 22.6 (3.3) | 22.3 (3.2) | 22.9 (3.4) |  |  |
| *Median (Q1, Q3)* | 22.0 (20.4, 24.6) | 21.6 (20.1, 24.0) | 22.7 (21.0, 25.2) |  |  |
| **BDI-II score** |  |  |  | 0.034 | d = 0.44 |
| *Mean (SD)* | 21.6 (7.9) | 23.3 (7.7) | 19.8 (7.8) |  |  |
| *Median (Q1, Q3)* | 21.0 (16.0, 26.0) | 23.0 (18.0, 27.0) | 18.0 (15.0, 23.0) |  |  |
| **HGSHS-5:G score** |  |  |  | <0.001 | d = -0.73 |
| *Mean (SD)* | 2.9 (1.6) | 2.3 (1.7) | 3.5 (1.3) |  |  |
| *Median (Q1, Q3)* | 3.0 (1.0, 4.0) | 2.0 (1.0, 4.0) | 4.0 (3.0, 5.0) |  |  |
| **CIS score** |  |  |  | <0.001 | d = -2.86 |
| *Mean (SD)* | 17.6 (7.7) | 11.4 (4.4) | 23.9 (4.3) |  |  |
| *Median (Q1, Q3)* | 18.5 (12.0, 22.0) | 12.0 (8.0, 15.0) | 22.0 (20.0, 27.0) |  |  |

Table S3: Baseline characteristics of the study sample stratified by suggestibility (HGSHS-5:G median split).

| **Characteristic** | **Overall (n = 94)** | **Low (n = 55)** | **High (n = 39)** | **p** | **Effect size** |
| --- | --- | --- | --- | --- | --- |
| **Age (years)** |  |  |  | 0.727 | d = -0.07 |
| *Mean (SD)* | 39.1 (12.1) | 38.7 (11.9) | 39.6 (12.5) |  |  |
| *Median (Q1, Q3)* | 37.5 (29.0, 49.0) | 37.0 (29.0, 48.0) | 39.0 (29.0, 51.0) |  |  |
| **Sex, n (%)** |  |  |  | 0.958 | V = 0.00 |
| *Female* | 67 (71.3%) | 39 (70.9%) | 28 (71.8%) |  |  |
| *Male* | 25 (26.6%) | 15 (27.3%) | 10 (25.6%) |  |  |
| *Diverse* | 2 (2.1%) | 1 (1.8%) | 1 (2.6%) |  |  |
| **Body mass index (kg/m²)** |  |  |  | 0.367 | d = -0.19 |
| *Mean (SD)* | 22.6 (3.3) | 22.3 (3.2) | 23.0 (3.5) |  |  |
| *Median (Q1, Q3)* | 22.0 (20.5, 24.6) | 21.7 (20.5, 24.0) | 23.1 (20.3, 25.4) |  |  |
| **BDI-II score** |  |  |  | 0.022 | d = 0.49 |
| *Mean (SD)* | 21.5 (7.9) | 23.1 (7.8) | 19.3 (7.6) |  |  |
| *Median (Q1, Q3)* | 21.0 (16.0, 26.0) | 22.0 (18.0, 27.0) | 18.0 (15.0, 24.0) |  |  |
| **HGSHS-5:G score** |  |  |  | <0.001 | d = -2.97 |
| *Mean (SD)* | 2.9 (1.6) | 1.7 (1.1) | 4.5 (0.5) |  |  |
| *Median (Q1, Q3)* | 3.0 (1.0, 4.0) | 2.0 (1.0, 3.0) | 4.0 (4.0, 5.0) |  |  |
| **CIS score** |  |  |  | 0.022 | d = -0.48 |
| *Mean (SD)* | 17.7 (7.7) | 16.1 (8.1) | 19.7 (6.7) |  |  |
| *Median (Q1, Q3)* | 19.0 (12.0, 22.0) | 15.5 (9.0, 22.0) | 20.0 (15.0, 24.0) |  |  |

Table S4: Correlations between baseline suggestibility and change in depressive symptoms from baseline to 6 weeks

| **Predictor** | **Group** | **N** | **r** | **95% CI** | **p** |
| --- | --- | --- | --- | --- | --- |
| HGSHS-5:G | Overall | 78 | -0.147 | [-0.358; 0.078] | 0.198 |
| HGSHS-5:G | Control | 30 | -0.201 | [-0.523; 0.172] | 0.287 |
| HGSHS-5:G | Hypnosis | 24 | 0.105 | [-0.311; 0.488] | 0.624 |
| HGSHS-5:G | PMR | 24 | -0.338 | [-0.652; 0.076] | 0.106 |
| CIS | Overall | 78 | -0.013 | [-0.235; 0.210] | 0.911 |
| CIS | Control | 30 | -0.194 | [-0.518; 0.179] | 0.304 |
| CIS | Hypnosis | 24 | 0.237 | [-0.184; 0.585] | 0.264 |
| CIS | PMR | 24 | -0.132 | [-0.509; 0.286] | 0.537 |

Table S5: Linear mixed-effects model (HGSHS-5:G)

| **Term** | **Estimate (β)** | **SE** | **df** | **t** | **95% CI** | **f²** | **p** |
| --- | --- | --- | --- | --- | --- | --- | --- |
| Intercept | 20.875 | 1.599 | 118.18 | 13.057 | [17.741; 24.008] | 1.443 | <0.001 |
| Time (T2 vs. T1) | -3.403 | 1.461 | 75.47 | -2.329 | [-6.266; -0.539] | 0.072 | 0.023 |
| HGSHS-5:G (z) | -3.642 | 1.599 | 117.89 | -2.277 | [-6.775; -0.508] | 0.044 | 0.025 |
| Hypnosis (vs. control) | 2.227 | 2.280 | 117.05 | 0.977 | [-2.242; 6.695] | 0.008 | 0.331 |
| PMR (vs. control) | -1.284 | 2.279 | 118.22 | -0.563 | [-5.752; 3.183] | 0.003 | 0.574 |
| Age (centered) | -0.021 | 0.069 | 84.58 | -0.299 | [-0.157; 0.115] | 0.001 | 0.766 |
| Male (vs. female) | 0.457 | 1.964 | 89.18 | 0.233 | [-3.393; 4.306] | 0.001 | 0.817 |
| Diverse (vs. female) | -0.589 | 6.035 | 96.35 | -0.098 | [-12.417; 11.239] | 0.000 | 0.922 |
| Time × HGSHS-5:G | -1.198 | 1.428 | 74.32 | -0.839 | [-3.996; 1.601] | 0.009 | 0.404 |
| Time × Hypnosis | -3.070 | 2.175 | 78.72 | -1.411 | [-7.333; 1.194] | 0.025 | 0.162 |
| Time × PMR | -1.663 | 2.153 | 77.13 | -0.773 | [-5.883; 2.556] | 0.008 | 0.442 |
| HGSHS-5:G × Hypnosis | 2.022 | 2.248 | 118.55 | 0.900 | [-2.384; 6.428] | 0.007 | 0.370 |
| HGSHS-5:G × PMR | 2.745 | 2.274 | 117.79 | 1.207 | [-1.711; 7.201] | 0.012 | 0.230 |
| Time × HGSHS-5:G × Hypnosis | 2.045 | 2.173 | 78.09 | 0.941 | [-2.214; 6.305] | 0.011 | 0.350 |
| Time × HGSHS-5:G × PMR | -0.966 | 2.127 | 77.08 | -0.454 | [-5.136; 3.204] | 0.003 | 0.651 |

Table S6: HGSHS model fit and diagnostics

| **Metric** | **Value** |
| --- | --- |
| N (participants) | 94 |
| AIC | 1180.70 |
| BIC | 1234.21 |
| logLik | -573.35 |
| Marginal R² | 0.186 |
| Conditional R² | 0.672 |
| ICC | 0.598 |
| Residual normality (p) | 0.824 |
| Homoscedasticity (p) | 0.738 |

Table S7: Linear mixed-effects model (CIS)

| **Term** | **Estimate (β)** | **SE** | **df** | **t** | **95% CI** | **f²** | **p** |
| --- | --- | --- | --- | --- | --- | --- | --- |
| Intercept | 21.867 | 1.633 | 115.55 | 13.387 | [18.666; 25.069] | 1.551 | <0.001 |
| Time (T2) | -2.963 | 1.426 | 74.65 | -2.078 | [-5.757; -0.169] | 0.058 | 0.041 |
| CIS (z) | -2.091 | 1.409 | 116.19 | -1.484 | [-4.852; 0.671] | 0.019 | 0.141 |
| Hypnosis (vs control) | 0.887 | 2.307 | 115.09 | 0.385 | [-3.634; 5.409] | 0.001 | 0.701 |
| PMR (vs control) | -2.514 | 2.338 | 116.09 | -1.075 | [-7.096; 2.068] | 0.010 | 0.284 |
| Age (centered) | -0.010 | 0.072 | 84.38 | -0.146 | [-0.151; 0.130] | 0.000 | 0.884 |
| Sex (male vs female) | 0.786 | 2.008 | 90.62 | 0.391 | [-3.150; 4.721] | 0.002 | 0.697 |
| Sex (diverse vs female) | -0.507 | 6.298 | 95.63 | -0.080 | [-12.850; 11.837] | 0.000 | 0.936 |
| Time × CIS | -0.944 | 1.226 | 73.72 | -0.770 | [-3.347; 1.459] | 0.008 | 0.444 |
| Time × Hypnosis | -3.350 | 2.111 | 77.43 | -1.587 | [-7.487; 0.788] | 0.033 | 0.117 |
| Time × PMR | -2.514 | 2.130 | 76.13 | -1.181 | [-6.688; 1.660] | 0.018 | 0.241 |
| CIS × Hypnosis | 0.758 | 2.323 | 116.09 | 0.326 | [-3.795; 5.310] | 0.001 | 0.745 |
| CIS × PMR | 2.910 | 2.232 | 116.44 | 1.304 | [-1.465; 7.284] | 0.015 | 0.195 |
| Time × CIS × Hypnosis | 3.061 | 2.256 | 78.90 | 1.357 | [-1.360; 7.482] | 0.023 | 0.179 |
| Time × CIS × PMR | -0.437 | 2.067 | 76.67 | -0.211 | [-4.488; 3.614] | 0.001 | 0.833 |

Table S8: CIS model fit and diagnostics

| **Metric** | **Value** |
| --- | --- |
| N (participants) | 94 |
| AIC | 1187.21 |
| BIC | 1240.72 |
| logLik | -576.61 |
| Marginal R² | 0.137 |
| Conditional R² | 0.673 |
| ICC | 0.622 |
| Residual normality (p) | 0.435 |
| Homoscedasticity (p) | 0.282 |

Figure S1: Distribution of baseline suggestibility and its association with change in depressive symptoms across treatment groups

*
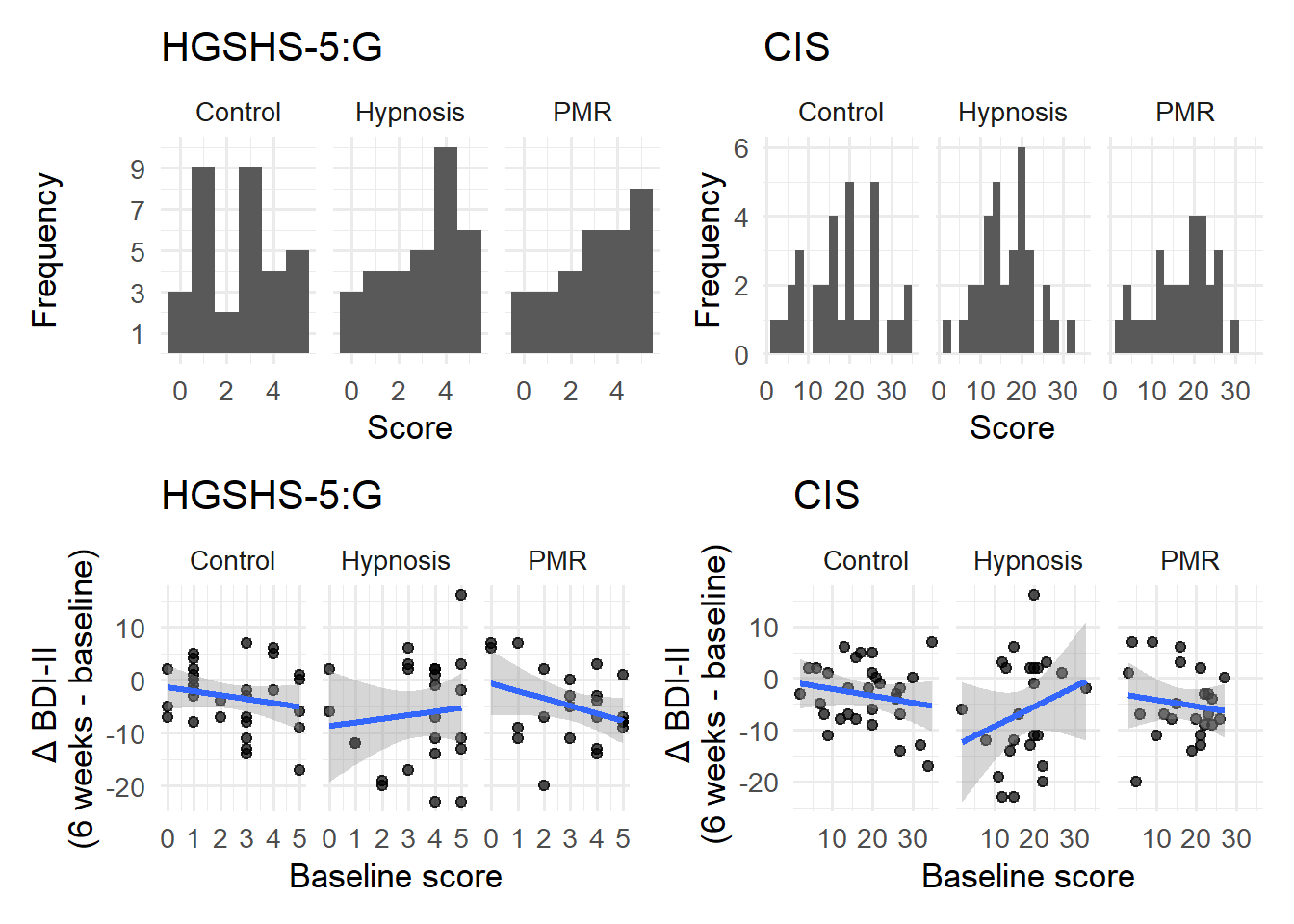
*
